# Autoimmune hepatitis in India: profile of an uncommon disease

**DOI:** 10.1186/1471-230X-5-27

**Published:** 2005-08-15

**Authors:** Gourdas Choudhuri, Sanjay K Somani, Chalamalasetty S Baba, George Alexander

**Affiliations:** 1 Department of Gastroenterology, Sanjay Gandhi Postgraduate Institute of Medical Sciences, Lucknow 226014 (UP), India

## Abstract

**Background:**

Autoimmune hepatitis (AIH) has been reported to show considerable geographical variation in frequency and clinical manifestations. It is considered a rare cause of liver disease in India. The present study was undertaken to determine the incidence, clinical, biochemical and histological profile of AIH in this part of the world.

**Methods:**

Patients presenting with acute or chronic liver disease between January 1999 and June 2002 were evaluated prospectively. AIH was diagnosed using the international autoimmune hepatitis group criteria. Workup included clinical, biochemical, USG, viral markers, UGI endoscopy, AI markers (ANA, SMA, Anti-LKM, AMA, RF, p-ANCA) using indirect immunofluorescence and liver biopsy if possible.

**Results:**

Forty-one of 2401 (1.70%) patients were diagnosed to have autoimmune liver disease. Out of these, 38 had autoimmune hepatitis and the rest 3 had primary biliary cirrhosis. The mean age of the patients of autoimmune hepatitis was 36.2 (15.9) years, 34 (89.4%) were females, and the duration of symptoms was 20.3 (20.5) months. Nineteen (50%) of them presented with chronic hepatitis, 13 (34.2%) as cirrhosis, 5 (13.1%) with acute hepatitis and 1 (2.6%) with cholestatic hepatitis. The presentations were jaundice in 21 (55.2%), pedal edema and hepatomegaly in 17 (44.7%), splenomegaly in 13 (34.2%), encephalopathy, abdominal pain in 9 (23.6%) and fever in 8 (21%). Twelve had esophageal varices and 3 had bled. Biochemical parameters were ALT 187 (360) U/L, AST 157 (193) U/L, ALP 246 (254) U/L, globulin 4.1 (1.6) g/dL, albumin 2.8 (0.9) g/dL, bilirubin 5.2 (7.4) mg/dL, prothrombin time 17 (7) sec and ESR 47 (17) sec. The autoimmune markers were SMA (24), ANA (15), both SMA and ANA (4), AMA (1), rheumatoid factor (2), pANCA (1), and Anti-LKM in none. Thirty (79%) patients had definite AIH and eight (21%) had probable AI hepatitis. Associated autoimmune diseases was seen in 15/38 (39.4%), diabetes 4, hypothyroidism 3, vitiligo 2, thrombocytopenia 2, rheumatoid arthritis 2, Sjogren's syndrome 1 and autoimmune polyglandular syndrome III in 1. Viral markers were positive in two patients, one presenting as acute hepatitis and HEV-IgM positive and another anti-HCV positive.

**Conclusion:**

In India, autoimmune hepatitis is uncommon and usually presents with chronic hepatitis or cirrhosis, acute hepatitis being less common. Age at presentation was earlier but clinical parameters and associated autoimmune diseases were similar to that reported from the west. Primary biliary cirrhosis is rare. Type II AIH was not observed.

## Background

Autoimmune hepatitis (AIH) is a disease of unknown etiology characterized by chronic hepatocellular inflammation, serum autoantibodies, and hypergammaglobulinemia, which in most cases respond to immunosupression [1-3]. Those affected are mainly young women. The course is generally progressive and often fluctuating and cirrhosis is often present when the disease is discovered.

The diagnosis of AIH is established by the revised scoring system devised by the International Autoimmune Hepatitis Group and the international association for the study of liver [4,5]. The overall sensitivity of the score to establish a diagnosis of definite or probable AIH was 89.8%, however, the specificity for discriminating AIH from overlapping syndrome such as PSC or PBC was low [6].

Histological studies show periportal hepatitis with lymphocytic infiltrates, plasma cells, and piecemeal necrosis. Lobular hepatitis can be present. Presence of granulomas and iron deposition argues against AIH [7,8].

Autoimmune hepatitis affects 100,000–200,000 individuals in United States [9]. In India the prevalence is less [10-13]. Some early reports have questioned the existence of autoimmune liver disease. The prevalence, nature and prognosis of autoimmune hepatitis remain unclear.

In this report, we have studied the frequency, clinical, biochemical and immunoserologic profile of autoimmune liver disease. We also have compared the acute and chronic presentation of autoimmune liver disease.

## Methods

### Study population

Consecutive patients with chronic or acute liver disease seen at a tertiary care center Sanjay Gandhi Postgraduate Institute of Medical Sciences, Lucknow, India from January 1999 to June 2002 were evaluated for etiology.

### Clinical assessment

In all patients, a detailed history was taken and clinical examination was carried out. History of onset of illness, acute or precipitating events, blood transfusion, surgery, menstrual abnormalities, and presence of extra-hepatic manifestations of autoimmune diseases were specifically noted. Family history of autoimmune diseases was also noted.

### Laboratory tests and virological assessment

All patients underwent biochemical evaluation using standard automated techniques. Liver function tests and serum proteins and serum globulins were done in all patients. HBsAg, HBeAg, Anti-HBe were measured by immunoenzymatic assays (enzyme-linked immunosorbant assays, Hepanostika, Organon Technika, Boxtel). Anti-HCV was done using a commercially available qualitative ELISA against peptides corresponding to highly antigenic segments of core, NS3, NS4, NS5 regions of hepatitis C (UBI HCV EIA 4.0, Beijing United Biomedical Co., Ltd., China). Patients who were anti-HCV positive were further tested for HCV-RNA using a RT-PCR technique. IgM-anti HEV immunoenzymatic assays (enzyme-linked immunosorbant assays, Hepanostika, Organon Technika, Boxtel) were also done in patients presenting with acute hepatitis.

### Immunoserologic assessment

Serological tests for autoantibodies antinuclear antibody (ANA), anti smooth muscle antibody (ASMA), anti-liver/kidney microsomal antibodies (anti-LKM-1) were done using the standard immunofluorescence technique. Briefly, cryostat sections of rat liver, kidney and stomach composite block were incubated with test sera (diluted 1:10) at 37°C for 30 min. After washing thrice with phosphate buffered saline (PBS) for 15 min each, the sections were incubated with fluoroscein isothiocyanate-conjugate rabbit anti-human polyclonal immunoglobulin (Dako, Copenhagen, Denmark) diluted 1:40 in PBS at 37°C for 30 min. The sections were washed thrice with PBS, mounted and examined under a fluorescent microscope. Titers of ANA, ASMA, and anti-LKM-1 of 1:80 or more is taken as positive in adults.

### Histological assessment

Percutaneous liver biopsy was done wherever possible. An experienced histopathologist examined all the liver biopsy and specifically looked for lymphoplasmacytic infiltrate, piecemeal or bridging necrosis, rosette formation, bile duct injury, siderosis, copper deposits and granulomas. Fibrosis was graded as periportal, bridging or complete regenerative nodules (cirrhosis).

Upper gastrointestinal endoscopy for evaluation of varices and portal hypertensive gastropathy was done using a videoendoscope in all patients (Pentax gastroscope, EG 2940, Japan). Endoscopic retrograde cholangiography was performed in patients with alkaline phosphate was elevated more than three fold of upper limit of normal with a clinical suspicion of primary sclerosing cholangitis. Real time ultrasound of upper abdomen was done in all patients to look for evidence of portal hypertension, collaterals, and ascites and to rule out any obstructive biliary pathology. Appropriate tests to exclude Wilson's disease and hemochromatosis were done wherever indicated.

Diagnosis of autoimmune hepatitis was based on the criteria of the International Autoimmune Hepatitis Group criteria (Table 1).[5] Diagnosis of Overlap syndrome was made if the patient had clinical, serological and histological characteristics of two conditions either at the same time or during the course of their illness. None of the patients of AIH had history of significant alcohol consumption, blood transfusion in the preceding 3 years, homosexual contact or intravenous drug abuse.

### Treatment

The indications for treatment were a greater than 2-fold elevation of aminotransferases in conjunction with interface hepatitis on liver biopsy. However in those in whom liver biopsy could not be done, elevation in liver enzymes alone was taken as indication for treatment. Response to treatment was defined as either or both of the following: marked improvement of symptoms and return of serum AST or ALT, bilirubin and immunoglobulin values completely to normal within 1 year and sustained for at least a further 6 months on maintenance therapy, or a liver biopsy showing at most minimal activity.

### Statistical analysis

Results are presented as mean (S.D.) and range. SPSS 10.0 was used for statistical analysis. Data in the two groups (acute and chronic presentations) was compared using Mann-Whitney U test. P-value <0.05 was taken as significant.

## Results

Of 2401 patients of liver disease who presented to the Gastroenterology department of Sanjay Gandhi Postgraduate of Medical Sciences, Lucknow, India, from January 1999 to June 2002, 41 (1.70%) patients fulfilled the International Autoimmune Hepatitis Group criteria [1]. Of these thirty-eight patients (1.5%) were confirmed to have autoimmune hepatitis and the other 3 had primary biliary cirrhosis based on liver biopsy findings. Thirty patients had definite (pretreatment score > 15) and eight had probable autoimmune hepatitis (pretreatment score 10–15) prior to treatment. But after initiation and continuation of treatment, 3 more attained scores of 17 and so were added to the definite autoimmune hepatitis group from the probable group. Among patients with autoimmune hepatitis, 35 (92.1%) were of type 1, none of type 2 and the rest 3 could not be classified as they lacked the SMA and ANA. The other etiologies of liver disease were hepatitis B in 30 %, hepatitis C in 20 %, alcohol in 25 %, cryptogenic in 15 % and others including autoimmune liver disease in 10 %.

**Figure 1:**
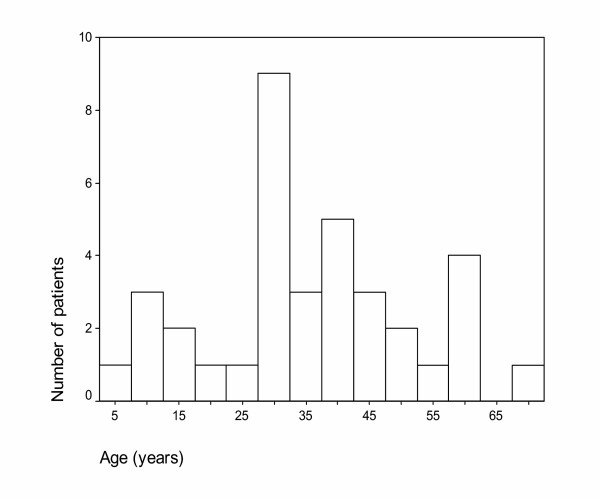
Age distribution of patients (n = 38)

Mean age of patients was 36.3 (2.6); range 7–68 years. Seven patients were less than 20 years and only five above 60 years of age. The age distribution at presentation showed a peak at 30 years (figure 1). There were 34 (89.4%) females; mean age of females [36.1 (16.7) range 7–68 years] was comparable with that of males [36.7 (13.8); range 11–51 years]. Mean duration of symptoms prior to presentation was 20.3 (3.9); range 0.2–72 months. Fifteen patients had duration of symptoms less than 6 months whereas 23 patients had chronic symptoms with duration of more than 6 months.

### Clinical findings

Nineteen (50%) patients presented with chronic hepatitis, thirteen (34.2%) with cirrhosis, five (13.1%) with acute hepatitis and one (2.6%) with cholestatic hepatitis. Jaundice, edema and fatigue were the most common clinical presentations. Jaundice was usually mild only. Physical findings showed ascites in more than one-third of patients. Table 2 shows the clinical parameters.

### Laboratory parameters

Mean alanine aminotransferase (ALT) was 187 (360) U/L and aspartate aminotransferase (AST) was 157 (193) U/L. Nine (22%) patients had AST and 11 (26.8%) had ALT above five times the upper limit of normal. The mean bilirubin was 5.2 mg/dl and nine patients (23.6%) had bilirubin above 5 mg/dL. See table 3 for the laboratory parameters.

### Upper gastrointestinal endoscopy

Esophageal varices were present in twelve (31.5%) patients of whom three had presented with bleed. Portal hypertensive gastropathy was present in three patients and gastric varices in two patients.

### Histological features

Liver biopsy was possible in only 19 (50%) patients, as 9 patients had coagulopathy, 6 had ascites and 4 had both ascites and coagulopathy, which contraindicated percutaneous liver biopsy. Chronic hepatitis was present in 15 and cirrhosis in 4. Interface hepatitis was present in 72.7%, rosette formation in 18.2% and lymphoplasmacytic infiltrates in 63.6%. Bile duct injury was observed in three patients.

### Virological markers

In the one patient tested positive for anti-HCV, the serum also tested positive for HCV-RNA by PCR. One other patient presented as acute hepatitis and tested positive for IgM-HEV. Patient improved clinically but continues to be SMA positive and has elevated transaminases. Acute hepatitis E led to the recognition of the underlying asymptomatic chronic autoimmune hepatitis. One patient had HIV ELISA positive and had chronic diarrhoea with partial villous atrophy on duodenal biopsy.

### Immunoserologic features

Antinuclear antibodies (ANA) was positive in 15 patients, of these 7 had speckled pattern (numerous evenly distributed specks of fluorescence, whereas remaining 8 had homogenous or diffuse pattern (uniform staining of nucleus). Anti-smooth muscle antibodies (SMA) were positive in 24 patients and out of these both ANA and SMA were positive in 4 patients. One of these patients had in addition AMA positivity and a high alkaline phophatase. But her liver biopsy did not reveal changes of PBC but showed features of autoimmune hepatitis (interface hepatitis and rosettes) and so she was classified under overlap syndrome (AIH-PBC). She had a score of 13, so falling under probable AIH. In 3 patients, no diagnostic antibodies were found and they were diagnosed on basis of other criteria including liver biopsy and response to immunosuppressive treatment. None of the patients had anti-liver/kidney microsomal (anti-LKM) antibodies.

### Associated autoimmune diseases

Fifteen (39.4%) patients had associated autoimmune disease (Table 5). Diabetes mellitus, autoimmune thyroiditis and vitiligo were the most common extra hepatic autoimmune features.

### Treatment outcomes

Thirty patients were started on treatment with corticosteroids (prednisolone) to which azathioprine was added in thirteen. In the remaining 8 patients, treatement with steroids was not started in 6 because of normal aminotransferases, presence of decompensated liver disease and liver biopsy (if done) not showing interface hepatitis and in another 2 because of being lost to follow up. Of the thirty patients started on steroids, 6 patients were lost to follow up and of the remaining 24, clinical and biochemical response was noticed in 17 (70.8 %). The patient with Overlap syndrome was treated with steroids but she did not achieve normalization of enzymes. Ursodeoxycholic acid was started in 5 patients in whom steroids either was not started or not tolerated, but only one patient achieved response. One patient on azathioprine had severe adverse effects in the form of pancytopenia and fever, requiring discontinuation of therapy. One patient had pulmonary tuberculosis in addition. He was on antitubercular drugs (isoniazid, rifampicin), on which he developed acute liver failure with hepatic encephalopathy and died due to increased intracranial tension. This patient was SMA positive. There were no other deaths in the study. One patient with decompensated cirrhosis who did not improve with immunosuppressant therapy underwent a liver transplant successfully and was doing well at 6 months follow-up.

Comparison of acute and chronic presentations did not show any significant difference between clinical, laboratory and immunoserologic parameters (Table 6). Histological parameters and treatment outcome also did not show any significant difference between these two groups.

## Discussion

The overall prevalence of autoimmune liver disease in our study was 1.70% and of autoimmune hepatitis, 1.50%. This contrasts with data from Western studies where the estimated prevalence is 11–20% of all cases of chronic liver disease [3,17]. This difference with the Western data has also been seen in other Indian studies, which have reported a low prevalence of 3.5–6.1% of all cases of chronic liver disease [10,13]. The low prevalence in our population may be attributed to possible genetic or geographic variation. Studies looking at genetic predisposing factors have been largely directed at genes of the immunoglobulin superfamily, which include those encoding HLA located in the MHC, immunoglobulins and T-cell receptor molecules. Geographic variations in these factors are seen, with type 1 AIH in Caucasians being associated with HLA-DR3 serotype while in Japan where HLA-DR3 is rare, the primary association is with HLA-DR4. Similar differences in these as well as other multiple genes might account for some of this low frequency of AIH seen in India and has to be studied.

Majority of the patients were in the third and fourth decades with a mean age of 36.3 (12.6) years and there was a female preponderance (F: M 4:1) seen. Although it has long been appreciated that AIH more affects girls and young and middle-aged women, it has been recognized that the disease is not uncommon in the elderly. In a series of patients with type 1 AIH in northern European Caucasians, elderly patients were not uncommon and the mean age of patients from a study from Japan was 50.8 (12.7) years [1,14]. In the present study only 5 patients (13.1%) were above 60 years of age and this observation of onset at a younger age in Indian patients has been seen in other Indian studies in which the reported mean ages have been 31.0 (17.1) and 39.8 (13) years with a female predominance [10,13]. The mean duration of symptoms at diagnosis in our study was 20.3, which was longer than that reported form other countries as well as from India [10].

Most of our patients had advanced liver disease at presentation. Cirrhosis was present in 34.2% of patients and chronic hepatitis in 50% patients. The onset of autoimmune hepatitis is usually insidious, as described in western studies with fatigue, fluctuating jaundice and arthralgia as typical features, but a substantial proportion of patients have no obvious signs or symptoms of liver disease or have an acute presentation as seen in 25 % [18]. In our study presentation as acute hepatitis accounted for only 13.1 % of all presentations. In patients with advanced disease liver biopsy was not possible in most cases due to complications like coagulopathy and ascites. The relative higher prevalence of chronic hepatitis may be misleading, as the true prevalence of cirrhosis could be higher and might be picked up only on liver biopsy.

In general, the elevations of aminotransferases are more striking than those of bilirubin and alkaline phosphatase. In some cases of AIH, however, a cholestatic picture is present, marked by high levels of conjugated bilirubin and alkaline phosphatase. In our study, mean bilirubin concentration was only 5.2 mg% and only 9 patients had bilirubin above 5 mg %. One patient presented with high levels of bilirubin and alkaline phosphatase with ANA, SMA and AMA positvity. Her liver biopsy was typical of AIH and so she was classified under cholestatic autoimmune hepatitis.

Type 1 autoimmune hepatitis was present in 92.1% patients and type 2 was not seen. Other Indian reports also show higher prevalence of type 1 AIH, 88.9% from Mumbai and 80% from Delhi [13,10]. It has been described that about 70 to 80 % patients of AIH present with significant titers of ANA or SMA (or both) and about 3 to 4 % have anti-LKM-1 antibodies, while up to 20 % have none of these antibodies [4]. In our study as previously described 92.1 % had SMA or ANA (or both) and in the rest no antibody could be identified. Diagnoses in these latter group of patients were made on basis of other criteria such as marked hypergammaglobulinemia, typical histological findings, immunogenetic background, family history of autoimmune diseases, appropriate investigations to exclude other possible causes of liver disease and response to immunosuppressive treatment. There is currently no agreement on what constitutes an autoimmune overlap syndrome. Two distinct types of overlap syndrome can be considered [20]. The crossover syndrome in which an individual may fit one diagnosis while having some features associated with another and the true overlap in which the patient has clinical, serologic and histologic characteristics of two conditions either at the same time or during the course of their illness. Only one patient in this study had the true overlap syndrome of AIH-PBC as she had cholestatic hepatitis with positivity of ANA as well as AMA, and liver biopsy features of AIH. In the other Indian study by Gupta et al also, a low frequency of overlap syndrome was seen in two patients [10].

Concurrent immunological diseases are reported in 17 to 48% of patients with autoimmune thyroiditis, synovitis and ulcerative colitis being most common [1-3,16]. We found it in 39.4%, with diabetes, thyroiditis and vitiligo being the most common. One patient had autoimmune polyglandular syndrome. Anti-HCV was positive in only one patient (2.8%) in whom the HCV-RNA was negative by PCR. A study from Japan, where hepatitis C has high prevalence, reported anti-HCV positivity in 12.5% of patients of autoimmune hepatitis [14]. Approximately 5 % of patients with chronic hepatitis C have ANA or SMA titers of 100 or higher and so in those patients where anti-HCV is positive, HCV-RNA has to be done also to rule out hepatitis C virus infection as the cause of the autoimmune phenomena [19].

Steroid monotherapy or combination therapy with prednisolone and azathioprine are the standard initial treatment of AIH. Combination therapy is best suited for elderly, osteoporotic patients, those with diabetes, hypertension, obesity and psychiatric disorders. Monotherapy with steroids is preferred in patients with hematological abnormalities and in young patients with fertility concerns. Ursodeoxycholic acid is a hydrophilic bile acid with immunomodulatary capability. Small-uncontrolled trials have shown clinical and biochemical improvement and a reduction in histological abnormality when given over 2 years [15]. Thirty of our patients received either prednisolone or azathioprine. Disease remission (clinical and biochemical) was achieved in seventeen patients. On the basis of limited data it has been shown that overlap syndromes of PBC or PSC and AIH with significant interface hepatitis may respond, at least in part to corticosteroids and such patients should receive a trial of immunosuppression [20]. The lone patient of overlap syndrome in the present study did not respond to a trial of steroids.

Comparison of acute and chronic presentation did not show any significant difference between clinical, laboratory, immunoserologic parameters, histology and treatment outcome. This indicates that autoimmune hepatitis of acute onset is in fact a chronic disease with abrupt onset of symptoms or acute exacerbation. The perceived acuteness or chronicity of the disease is a reflection of detection bias. Due to a long duration of sub clinical disease, it may be irrational to distinguish acute and chronic autoimmune hepatitis as both the presentations have similar clinical, biochemical, immunoserologic and histologic features.

## Conclusion

In summary, our patients presented at an early age, had a longer duration of symptoms. Female preponderance was observed. Type 1 autoimmune hepatitis was the most common, whereas type 2 was not observed in our patients. Clinical presentation was with advanced liver disease (chronic hepatitis and cirrhosis) and acute hepatitis was less common. Most patients had SMA or ANA positive. Associated autoimmune diseases were common. Hepatitis C infection was uncommon in our patients. Comparison of acute and chronic presentations did not reveal any significant difference, questioning the need for any such distinction.

## Abbreviations

ANA: anti-neutrophilic antibody

AMA: anti-mitochondrial antibody

Anti-LKM: anti-liver/kidney microsomal antibodies

p-ANCA: anti-neutrophil cytoplasmic antibody

RF: rheumatoid factor

PBC: primary biliary cirrhosis

PSC: primary sclerosing cholangitis

## Competing interests

The author(s) declare that they have no competing interests.

## Contributions

GC: conceived the study, participated in its design, coordination and drafting the manuscript

SKS, GA: participated in the study design, collection of data and drafting the manuscript

CSB, TSN: participated in the study design and collection of data

## Pre-publication history

The pre-publication history for this paper can be accessed here:



## Figures and Tables

**Table 1:** International diagnostic criteria for diagnosis of AIH

Parameter	Score
Gender	
Female	+2
Male	0

Serum biochemistry	
Ratio of elevation of serum alkaline phosphatase vs. aminotransferase	
>3.0	-2
1.5–3	0
<1.5	+2

Total serum globulin, γ-globulin, or IgG (times upper limit normal)	
>2.0	+3
1.5–2.0	+2
1.0–1.5	+1
<1.0	0

Autoantibodies (titers by immunofluorescence on rodent tissue)	
ANA, SMA, or LKM-1	
>1:80	+3
1:80	+2
1:40	+1
<1:40	0

Antimitochondrial antibodies	
Positive	-4
Negative	0

Hepatitis viral markers	
Negative	+3
Positive	-3

Other etiological factors	
History of drug usage	
Yes	-4
No	+1
Alcohol (average consumption)	
<25 g/day	+2
>60 g/day	-2
Genetic factors: HLA DR3 or DR4	+1
Other autoimmune diseases	+2

Response to therapy	
Complete	+2
Relapse	+3

Liver histology	
Interface hepatitis	+3
Predominant lymphoplasmacytic infiltrate	+1
Rosetting of liver	+1
None of above	-5
Biliary changes	-3
Other changes	-3
Seropositivity for other defined autoantibodies	+2

Definite AIH, >15 before treatment and >17 after treatment; probable AIH 10–15 before treatment and 12–17 after treatment

**Table 2:** Clinical features (n = 38)

Clinical features	No (%)
Symptoms	
Jaundice	21 (55.2)
Edema	17 (44.7)
Fatigue	17 (44.7)
Encephalopathy	9 (23.6)
Pruritus	9 (23.6)
Abdominal pain	9 (23.6)
Fever	8 (21.0)
Arthralgia	7 (18.4)
Menstrual abnormalities	4 (10.5)
	
Physical signs	
Hepatomegaly	17 (44.7)
Splenomegaly	13 (34.2)
Ascites	13 (34.2)
Vitiligo	3 (7.8)

**Table 3:** Lab parameters (n = 38)

Parameters	Mean ± SD	Range	% with abnormal value
Bilirubin (mg/dl)	5.2 ± 7.4	(0.21–33.4)	63.9
ALT (U/L)	187 ± 360	(10–2100)	75.0
AST (U/L)	157 ± 193	(10–986)	77.8
ALP (U/L)	230 ± 230	(63–935)	41.7
Albumin (g/dL)	2.8+0.9	(1.2–4.3)	75.0
Globulin (g/dL)	4.6+1.5	(2.0–8.1)	58.3
ESR (sec)	45+18	(12–72)	55.0
Prothrombin time (sec)	17+7	(8–36)	50.0

**Table 4:** Autoimmune markers (n = 38)

Autoimmune marker	n	%
SMA	24	(63.1)
ANA	15	(39.4)
SMA + ANA	4	(10.5)
AMA	1	(2.6)
p-ANCA	1	(2.6)
Rheumatoid factor	2	(5.2)

**Table 5:** Extra hepatic manifestations

Associated autoimmune diseases	n
Diabetes	4
Thyroiditis	3
Vitiligo	2
Thrombocytopenia	2
Rheumatoid arthritis	2
Sjogren's syndrome	1
AI polyglandular syndrome type III	1

**Table 6:** Initial findings in autoimmune hepatitis with acute and chronic presentations

Initial findings	Acute presentation (n = 15)	Chronic presentation (n = 23)
Clinical		
Age (years)	36.6 (16.5)	36.0 (16.0)
Sex (F: M)	13:3	4:1
Laboratory parameters		
ALT (10–40 U/L)	143 (139)	213 (457)
AST (10–40 U/L)	187 (247)	207 (393)
ALP (U/L)	209 (174)	251 (280)
Bilirubin (0.2–0.8 mg/dL)	7.3 (9.6)	3.6 (4.7)
Albumin (3.5–4.5 g/dL)	2.6 (0.9)	3.1 (0.8)
Globulin (2.5–3.5 g/dL)	4.8 (1.8)	4.5 (1.4)
Prothrombin time (sec)*	19 (8.2)	15.9 (4.5)
Immunoserologic markers		
ANA n (%)	6 (40.0%)	9 (39.1%)
SMA n (%)	9 (60.0%)	15(65.2%)
AMA n (%)	0	1 (4.3%)

Values expressed as Mean (S.D.)

* Control 12.7 sec

Comparison of parameters in the two groups did not show any statistical difference.
